# Meteorological Table

**Published:** 1849-07

**Authors:** John Lea


### METEOROLOGICAL TABLE.

CINCINNATI, May, 1849.

| Thermometer. | | | Weather. | | | |
|---|---|---|---|---|---|---|
| Date. | Minimum. | Maximum, | Sunrise. | Noon. | Sunset. | Rain. |
| 1 | 518 | 639 | rain and clear | clear | clear | 00.05 |
| 2 | 48 | 70 | clear | clear | clear | |
| 3 | 55 | 85 | clear | clear | variable | |
| 4 | 68 | 83 | clear | clear | clear and rain | .10 |
| 5 | 67 | 73 | variable | rain | rain | 1.95 |
| 6 | 68 | 79 | cloudy and rain | clear | clear | .05 |
| 7 | 65 | 84 | clear | clear | var. and rain | .40 |
| 8 | 63 | 75 | clear | variable | variable | |
| 9 | 58 | 74 | clear | clear | clear | |
| 10 | 52 | 65 | variable | variable | clear | |
| 11 | 48 | 71 | clear | clear | clear | |
| 12 | 54 | 69 | cloudy | variable | var. and rain | .55 |
| 13 | 55 | 64 | clear | clear | clear | |
| 14 | 53 | 72 | clear | clear | clear | |
| 15 | 50 | 72 | cloudy | variable | clear | |
| 16 | 57 | 74 | rain and clear | clear | clear | .05 |
| 17 | 53 | 78 | clear | clear | variable | |
| 18 | 57 | 77 | clear | clear | variable | |
| 19 | 55 | 79 | clear | clear | clear | |
| 20 | 58 | 70 | clear | clear | clear | |
| 21 | 64 | 83 | clear | clear | cloudy and rain | .25 |
| 22 | 65 | 82 | var. and rain | clear | clear | .10 |
| 23 | 62 | 78 | clear | clear | clear | |
| 24 | 56 | 69 | clear | clear | clear | |
| 25 | 47 | 66 | clear | clear | clear | |
| 26 | 50 | 77 | clear | clear | var and rain | |
| 27 | 61 | 73 | cloudy | clear | variable | |
| 28 | 59 | 70 | cloudy | variable | cloudy and rain | .15 |
| 29 | 57 | 67 | cloudy | variable | rain and clear | .15 |
| 30 | 56 | 76 | fog and var- | clear | clear | |
| 31 | 59 | 79 | fog and clear | clear | clear | |
| | | | | | Inches | 3.80 |

| | | | |
|---|---|---|---|
| Mean temperature of the month | | | 65.74 |
| Do | do | May, 1848 | 68.55 |
| Do | do | May, 1847 | 64.26 |
| Do | do | May, 1846 | 69.48 |
| Do | do | May, 1845 | 63.23 |
| Do | do | May, 1844 | 67.73 |
| Do | do | May, 1843 | 63.22 |
| Clear days in the month | | | 11 |
| Variable (clear at times) | | | 20 |
| Cloudy (sun not visible) | | | 00—31 |

*Remarks.*—Mean temperature of the month about the usual average. Prevailing wind westerly; a portion of seven days easterly, and calms on part of seven days; light winds on part of 23 days; brisk breezes on a portion of eight days, and stormy part of the nights of the 12th and 13th.

### METEOROLOGICAL TABLE.

CINCINNATI, June, 1849.

| Thermometer. | | | Weather. | | | | Remarks, Winds, &c. |
|---|---|---|---|---|---|---|---|
| Date. | Minimum. | Maximum. | Sunrise. | Noon. | Sunset. | Rain. | |
| 1 | 59° | 84° | clear | clear & rain | clear | 00.15 | Light S westerly, squally, S westerly, calm. |
| 2 | 64 | 84 | clear | clear | clear | | " southerly. |
| 3 | 65 | 88 | clear | clear | clear | | " S westerly. |
| 4 | 67 | 90 | clear | clear | clear | | " " and variable. |
| 5 | 63 | 85 | clear | clear | clear | | " N easterly, calm at night. |
| 6 | 65 | 79 | clear | clear | var. & rain | 00.10 | " easterly and light S easterly. |
| 7 | 69 | 86 | clear | clear | rain & clear | 00.20 | Light southerly and calm, |
| 8 | 74 | 87 | cloudy and rain | clear | var. & rain | 00.80 | Calm, light westerly, and calm. |
| 9 | 68 | 78 | cloudy | clear and rain | clear | 00.05 | " light northerly, and calm. |
| 10 | 61 | 79 | var. and rain | clear | clear | 00.10 | Light S easterly and calm. |
| 11 | 64 | 80 | clear and rain | clear | clear | 00.10 | " " " " |
| 12 | 68 | 84 | cloudy | variable | clear | | " southerly and S westerly. |
| 13 | 70 | 89 | variable | clear | variable | | " southerly brisk southerly P. M. |
| 14 | 72 | 89 | variable | clear | var. & rain | 00.20 | " " " S westerly P. M. |
| 15 | 70 | 73 | clear | clear | clear | | " S westerly and westerly, shower. |
| 16 | 62 | 79 | clear | clear | clear | | " N westerly, calm at evening. |
| 17 | 61 | 75 | clear | clear | clear | | Calm and light southerly. |
| 18 | 62 | 88 | clear | clear | clear | | " " " and calm. |
| 19 | 68 | 89 | clear | clear | clear | | Light southerly and calm. |
| 20 | 69 | 93 | clear | clear | clear | | Calm, very light southerly, and calm. |
| 21 | 70 | 92 | clear | clear | clear | | " light southerly and calm. |
| 22 | 72 | 93 | clear | clear | clear | | " and brisk southerly, calm at night. |
| 23 | 75 | 87 | clear | clear | clear & rain | 00.05 | " and light southerly. |
| 24 | 73 | 84 | cloudy | clear and rain | variable | 00.65 | " " " brisk S w'ly, heavy thunder and calm. |
| 25 | 72 | 83 | fog and var. | cloudy | rain & clear | 00.15 | " light southerly and calm. |
| 26 | 74 | 90 | cloudy | clear | clear | | " " " |

| | | | | | | | |
|---|---|---|---|---|---|---|---|
| 27 | 74 | 87 | clear | clear and rain | var. & rain | 00.35 | Calm light S easterly, westerly and calm. |
| 28 | 73 | 80 | clear | clear and rain | var. & rain | 00.40 | Light southerly, brisk westerly, thunder, P. M. |
| 29 | 72 | 87 | clear and rain | clear & rain | clear & rain | 1.25 | Calm and southerly, thunder, calm. |
| 30 | 72 | 88 | cloudy | variable | variable | | " and southerly, N west'ly P. M. and calm, thunder at night. |

| | |
|---|---|
| Rain Inches | 4.55 |
| Clear days in the month | 12 |
| Variable | 18 |
| Cloudy (total absence of sun) | 00 |
| | 30 |
| Mean temperature of the month | 76.96 |
| Do do of June, 1848 | 72.14 |
| Do do of June, 1847 | 70.36 |
| Do do of June, 1846 | 69.84 |
| Do do of June, 1845 | 74.00 |
| Do do of June, 1844 | 73.20 |
| Do do of June, 1843 | 70.77 |

| | | |
|---|---|---|
| Max. heat of the sun on the 22nd | | 121° |
| " " " | 26th | 122 |

There were other days in the month, in which the sun was probably at 125°

*Remarks.*—Rains are more frequent than is usual for June, but less in quantity. The mean temperature of the month is higher than usual.

An unusual proportion of calms and light winds, rendering the few hot days more oppressive.

The thermometer from which these observations are recorded hangs on the north side of the house, in a portico, behind a pillar, to prevent the reflection of the sun's rays affecting it. The course of winds is taken from the direction of smoke from chimnies, as the vanes cannot be depended on in light winds.

Cincinnati, July, 1849.

| | | | | | | | |
|---|---|---|---|---|---|---|---|
| 1 | 69 | 84 | rain | clear | clear | 2.35 | Light S easterly. |
| 2 | 64 | 78 | clear | clear | clear | | " northerly, brisk easterly, calm at night. |
| 3 | 64 | 81 | clear | clear | clear | | Brisk easterly, light do at evening. |
| 4 | 61 | 82 | clear | clear | clear | | " " " do calm at evening. |
| 5 | 61 | 82 | clear | clear | clear | | Light south. brisk P. M., light at evening. |
| 6 | 68 | 69 | drizzle | rain | rain | 1.10 | " " " calm at night. |
| 7 | 67 | 77 | cloudy | var. | rain and clear | .30 | " " and S west. " " |
| 8 | 71 | 83 | cloudy | var. | var. | | Calm and light south, S east. and calm, heavy thunder. |
| 9 | 76 | 91 | rain and clear | clear | clear | 2.45 | " " S west and west. calm at night, thunder. |
| 10 | 77 | 90 | rain and var. | clear | clear | .15 | Calm and light south, calm at evening. |
| 11 | 75 | 92 | clear | clear | var. and rain | .10 | Light S east. " " |
| 12 | 71 | 90 | clear | clear | clear | | " south. |
| 13 | 74 | 92 | clear | clear | clear | | " " calm at evening and night. |
| 14 | 76 | 92 | variable | clear | clear | | Light and north. |

| Thermometer. | | | Weather. | | | | |
|---|---|---|---|---|---|---|---|
| Date. | Minimum. | Maximum. | Sunrise. | Noon. | Sunset. | Rain. | Winds, &c. |
| 15 | 64 | 83 | clear | clear | clear | | Light N west. calm at evening. |
| 16 | 63 | 85 | " | " | " | | " S east. " " |
| 17 | 64 | 84 | " | " | rain and clear | .10 | Calm and light south. |
| 18 | 67 | 88 | fog and clear | " | clear | | " very " " |
| 19 | 69 | 88 | " | " | clear and rain | .15 | " and " " |
| 20 | 74 | 85 | cloudy and rain | var. | clear | .05 | " and brisk, S west. calm at night. |
| 21 | 73 | 83 | var. | clear | " | | " and light west. brisk N west. |
| 22 | 65 | 82 | clear | " | " | | Light east. |
| 23 | 64 | 86 | " | " | " | | Light N east. brisk east. and calm. |
| 24 | 67 | 82 | var. | cloudy | rain | 1.15 | Light S. east. |
| 25 | 72 | 83 | cloudy and rain | var. | rain | .40 | Brisk south. |
| 26 | 71 | 88 | clear | clear | rain and clear | .15 | Light N west. |
| 27 | 70 | 84 | var. | " | clear | | Calm A. M., light S east, P. M., |
| 28 | 70 | 87 | clear | " | " | | Light and south. calm at night. |
| 29 | 70 | 85 | " | clear and rain | " | .15 | " " A. M., brisk S W P. M., calm at evening. |
| 30 | 76 | 82 | clear and rain | var. | " | .40 | " S west. (heavy thunder) and west. |
| 31 | 60 | 77 | clear | clear | " | | " N east. calm at night. |
| | | | | Rain Inches | | 9.00 | |

Clear days in the month 13; Variable (cloudy at times) 17; Cloudy (total absence of sun) 1.

Max. heat of the sun on the 3rd 121°; on the 11th, 128°; on the 13th 125°; on the 21st 114°; on the 26th 124°.

Mean temperature of the month 76.42; do. of July, 1848, 73.86; do. of July, 1847, 74.42; do. of July, 1846, 76.00; do. of July, 1845, 75.84; do. of July, 1844, 78.47; do. of July, 1843, 75.10.

*Remarks.*—Mean temp. of the month rather over the usual average.

Quantity of rain considerably over the usual average; yet there was but one day in which the sun was not visible. Calms have been frequent throughout the month, and no high wind has occurred.

The greatest number of deaths by Cholera (137) occurred on the 5th July.

